# Dose distributions of proton therapy plans are robust against lowering the resolution of CTs combined with increasing noise

**DOI:** 10.1002/mp.17530

**Published:** 2024-11-28

**Authors:** Carla Frensch, Claus Maximilian Bäcker, Walter Jentzen, Ann‐Kristin Lüvelsmeyer, Mohammadreza Teimoorisichani, Jörg Wulff, Beate Timmermann, Christian Bäumer

**Affiliations:** ^1^ West German Proton Therapy Centre Essen Essen Germany; ^2^ West German Cancer Center (WTZ) University Hospital Essen Essen Germany; ^3^ Department of Physics TU Dortmund University Dortmund Germany; ^4^ Clinic for Nuclear Medicine University Hospital Essen Essen Germany; ^5^ Siemens Medical Solutions USA Inc. Knoxville Tennessee USA; ^6^ German Cancer Consortium (DKTK) Essen Germany; ^7^ Department of Particle Therapy University Hospital Essen Essen Germany

**Keywords:** CT dose reduction, image quality, proton therapy, robust optimization, spatial resolution

## Abstract

**Background:**

Treatment planning in radiation therapy (RT) is performed on image sets acquired with commercial x‐ray computed tomography (CT) scanners. Considering an increased frequency of verification scans for adaptive RT and the advent of alternatives to x‐ray CTs, there is a need to review the requirements for image sets used in RT planning.

**Purpose:**

This study aims to derive the required image quality (IQ) for the computation of the dose distribution in proton therapy (PT) regarding spatial resolution and the combination of spatial resolution and noise. The knowledge gained is used to explore the potential for dose reduction in tomography‐guided PT.

**Methods:**

Mathematical considerations indicate that the required spatial resolution for dose computation is on the scale of the set‐up margins fed into the robust optimization. This hypothesis was tested by processing retrospectively 12 clinical PT cases, which reflect a variety of tumor localizations. Image sets were low‐pass filtered and were made noisy in a generic manner. Dose distributions on the modified CT scans were computed with a Monte‐Carlo dose engine. The similarity of these dose distributions with clinical ones was quantified with the gamma‐index (1 mm/1%). The potential reduction of the x‐ray exposure compared to the planning CT scan was estimated.

**Results:**

Dose distributions within the irradiated volume were robust against low‐pass filtering of the CTs with kernels up to a full‐width‐at‐half‐maximum of 4 mm, that is, the gamma pass rate (1 mm/1%) was ≥98%. The limit of the filter width was 6 mm for brain tumors and 8 mm for targets in the abdomen. These pass rates remained approximately unchanged if a limited amount of noise was added to the CT image sets. The estimated potential reductions of the x‐ray exposure were at least a factor of 20.

**Conclusions:**

The requirements on IQ in terms of spatial resolution in combination with noise for computing the dose in PT are clearly lower than the IQ of current clinical planning. The results apply, for example, to ultra‐low dose x‐ray CTs, proton CTs with coarse spatial detection, and attenuation images from the joint reconstruction of time‐of‐flight PET scans.

## 1 INTRODUCTION

Computed tomography (CT) image sets serve many purposes in radiation therapy (RT). Besides segmentation, image‐guided patient positioning, response assessment and adaptive treatment planning,^1^ CTs are used for dose computation and optimization of treatment plans. Each of above tasks has its own requirements on image quality (IQ) such us geometric accuracy and contrast. While the virtual treatment simulation for proton therapy (PT) has been conducted with x‐ray CTs for decades, the concept of a proton CT^2^, ^3^ has been realized in recent research projects.^4^, ^5^, ^6^ Furthermore, synthetic CTs generated from magnetic resonance imaging (MRI)^7^ and γCTs from a joint reconstruction of time‐of‐flight (ToF) positron emission tomography (PET) acquisitions^8^ are being researched as potential modalities for future image guidance in PT. These imaging modalities are potentially associated with inferior IQ.

The current study seeks to explore the quality requirements of tomographic image sets for dose computation in PT in terms of spatial resolution combined with noise. The insights gained were used to point out options for the reduction of dose expended for tomographic imaging in PT. This study focuses on the dosimetric impact of image sets whose spatial resolution is lower than usual in current clinical PT planning. Clinical x‐ray CTs were retrospectively degraded in a generic manner in terms of spatial resolution and noise. The modified image sets were subsequently evaluated in terms of suitability for dose computation. The robustness of proton fields against noise on the planning CTs was shown first by Chvetsov and Paige^9^ and elaborated later for PT plans by Elhamiasl et al.,^10^ who processed the scans in the projection domain thereby accounting for the physics and technique of a commercial x‐ray CT scanner.^11^

Section 2 formulates the theoretical expectation that the scale of the required spatial resolution corresponds to the set‐up margin fed into the robust optimization of a treatment plan. Section 3 investigates the requirements in an independent manner, namely through a retrospective in‐silico study. The results of the in‐silico evaluation are presented in Section 4. The discussion (Section 5) compares the theoretical result with the outcome of the in‐silico investigation, points out applications in PT, and considers strategies for dose reduction.

## 2 THEORETICAL CONSIDERATIONS

A CT based map $\tilde{c}\left(\vec{x}\right)$ serves as a patient model for dose computations in RT. This map provides for each voxel $\vec{x}=\left(x,y,z\right)$ a gray value in terms of Hounsfield units (HU), an electron density $n_{e}$, mass density ρ or a stopping‐power ratio. The minimum spatial resolution of $\tilde{c}$ for dose computation in RT shall be estimated in the following. An ideal map $\tilde{c}$ is assumed, which is approximated by clinical scans for virtual simulation, and compared with a low‐resolution (LR) map. The relation between the ideal map and the LR map can be described by the folding operation with a kernel p:

(1) $${\tilde{c}}_{\mathrm{LR}}\left(x\right)=\tilde{c}*p=\int_{-\infty }^{\infty }p\left(s\right)\tilde{c}\left(x-s\right)ds$$

which is restricted to a single dimension for simplicity. In this study, a normal distribution (Gaussian), which corresponds to the usual approximation of a point‐spread function (PSF) of x‐ray CTs, and a boxcar‐type filter were used as kernel p. The latter type of filter corresponds to the shape of the set‐up margin, which is uniform with a hard cutoff and theoretically related to the spatial resolution of a map as will be developed in the course of this section. The right hand side of Equation (1) can be interpreted as a two‐step operation comprising the generation of a perturbed scenario of the virtual patient by shifting by s and the weighted addition of the perturbed scenarios. Later, the shift s is identified with the lateral shift used in robust optimization and robust evaluation. We change to a voxel representation $c_{k}$ $\left(k=1..N^{\prime }\right)$ of $\tilde{c}$ ‐ and similarly for ${\tilde{c}}_{\mathrm{LR}}$—with voxel width Δv. Equation (1) transforms to

(2) $$c_{\mathrm{LR},k}=\sum_{j}p\left(j\Delta v\right)c_{k-j}$$

The definition of $c_{k}$ in one dimension can be extended to three dimensions by numbering the voxels from 1 to N, which is the product of $N^{\prime }$ and the number of voxels in the other two dimensions. Suppose we have a function $D_{i}$, which calculates the absorbed dose in voxel i. This function is similar to the notion of a response function in ref. 12. A treatment plan will fulfill

(3) $$D_{i}\left(c_{1},c_{2},\text{…},c_{N}\right)=D_{\mathrm{prescr}}$$

for voxels i of a target ROI, for example, the clinical target volume (CTV). Similar to a LR CT, the incorporation of set‐up uncertainties $\left(u_{x},u_{y},u_{z}\right)$ also induces some fuzziness in the spatial domain. If the dose distribution is insensitive to it, then the treatment plan is robust,^13^ that is, dose voxels i of the CTV fulfill:

(4) $$D_{i}\left(c_{1}\left(s\right),\text{…},c_{N}\left(s\right)\right)=D_{\mathrm{prescr}}$$

where the considerations are restricted to a setup shift s in a single cardinal direction:

(5) $$c_{k}\left(s\right)=\tilde{c}\left(k\Delta v-s\right)\;\;\mathrm{with}\;\;s<u_{x}/y/z\;\;\mathrm{and}\;\;k=1..N^{\prime }$$

Note that this equation corresponds to an ideal scenario. In practice, the = in Equation (4) should be replaced by ≈ as, for example, dose variations of ±5% are usually accepted in the target volume.

Differentiation of Equation (4) yields:

(6) $$\begin{array}{c} \frac{dD_{i}\left(c_{1}\left(s\right),\text{…},c_{N}\left(s\right)\right)}{ds}=\sum_{k}\frac{\partial D_{i}\left(c_{1}\left(s\right),\text{…},c_{N}\left(s\right)\right)}{\partial c_{k}}\frac{\partial c_{k}\left(s\right)}{\partial s}=0 \end{array}$$

Now the example of a perturbed scenario $c_{j}\left(s\right)$ and its weighted averaging with the nominal scenario $c_{j}$ is considered:

(7) $$\begin{array}{c} c_{j}^{\prime }=\left(1-w\right)c_{j}+wc_{j}\left(s\right)=c_{j}+\Delta c_{j}\;\mathrm{with}\;\Delta c_{j}=wc_{j}\left(s\right)-wc_{j} \end{array}$$

where w (0<w<1) is the relative weight.

(8) $$\Delta c_{j}=-wc_{j}\left(0\right)+wc_{j}\left(0\right)+w\frac{dc_{j}\left(0\right)}{ds}s+O\left(s^{2}\right)$$

We would like to know the corresponding dose distribution, which is approximated by a Taylor series:

(9) $$\begin{array}{ccc} D_{i}\left(c_{1}^{\prime },\text{…},c_{N}^{\prime }\right) & = & D_{i}\left(c_{1},\text{…},c_{N}\right)\\ & & +\sum_{k}\frac{\partial D_{i}\left(c_{1},..,c_{N}\right)}{\partial c_{k}}\Delta c_{k}+O\left(\Delta c_{k}^{2}\right)\; \end{array}$$

With Equation (8) this equation transforms to:

(10) $$\begin{array}{ccc} D_{i}\left(c_{1}^{\prime },\text{…},c_{N}^{\prime }\right) & = & D_{i}\left(c_{1},..,c_{N}\right)\\ & & +\;w\sum_{k}\frac{\partial D_{i}\left(c_{1},..,c_{N}\right)}{\partial c_{k}}\frac{\partial c_{k}}{\partial s}s+O\left(s^{2}\right)\; \end{array}$$

The second addend is zero (small) according to Equation (6). Thus, the dose remains approximately constant for a linear combination of a density map ${\tilde{c}}_{k}$ and its shifted copy ${\tilde{c}}_{k}\left(s\right)$ if the spatial shift s is within the robust setup criterion $u_{x}$:

(11) $$D_{i}\left(c_{1}^{\prime },\text{…},c_{N}^{\prime }\right)=D_{i}\left(c_{1},..,c_{N}\right)+O\left(s^{2}\right)$$

We extend the scenario of the superposition of density maps $c_{k}$ with relative shift s (Equation 7) by a superposition of a multitude of density maps (convolution in Equation 2) and get with Equation (10) for the dose

(12) $$\begin{array}{ccc} D_{i}\left(c_{1}^{\prime },\text{…},c_{N}^{\prime }\right) & = & D_{i}\left(c_{1},..,c_{N}\right)\\ & & +\;\sum_{j\neq 0}p\left(j\;s\right)\left(\sum_{k}\frac{\partial D_{i}\left(c_{1},..,c_{N}\right)}{\partial c_{k}}\frac{\partial c_{k}}{\partial s}\right)js+\cdots \end{array}$$

This gives the same result as Equation (11). Therefore, in a first‐order approximation the dose remains constant if the underlying image set is low‐pass filtered with filter widths smaller than the set‐up margin.

Above theoretical considerations refer to sub‐volumes of uniform dose, which have been planned with a robust optimization. The extent to which this applies to normal tissue, which is covered by an almost uniform dose, is assessed below. Sub‐volumes within a dose gradient are not covered by the theory. Let's consider organs‐at‐risk (OAR). Similar to Equation (4) we require for voxels i within an OAR

(13) $$D_{i}\left(c_{1}\left(s\right),\text{…},c_{N}\left(s\right)\right)\leq D_{\mathrm{OAR}}$$

Equation (13) applies to the case that the dose burden is clearly below the tolerance dose $D_{\mathrm{OAR}}$ and could comprise, for example, a sharp dose gradient within the OAR. The derivative (Equation 6) is limited in Equation (13). This limits the first‐order term in Equation (10), but a quantitative statement if the OAR constraint is met cannot be made. As an alternative to Equation (13), one may require

(14) $$D_{i}\left(c_{1}\left(s\right),\text{…},c_{N}\left(s\right)\right)\leq D_{i}\left(c\left(0\right)\right)$$

As a clinical example, Equation (14) refers to the case that the dose burden of an OAR is already at the tolerance level and any dose increase must be avoided in perturbed irradiation scenarios. In case of Equation (14), the derivative (Equation 6) is zero or negative. Thus, Equation (10) holds in first order. Summarizing the considerations about OAR tolerances, the mathematical evidence is less conclusive in the sense that the properties of a treatment plan imply robustness against the CT resolution in the frame of dose computation. However, the equations provide indications for limited effects on the dose distribution, especially for OAR dose burdens at the tolerance limit.

## 3 MATERIALS AND METHODS

Twelve clinical cases were evaluated retrospectively. All patients selected for evaluation were enrolled in two prospective registries ProReg (Registry number: DRKS00004384) and KiProReg (Registry number: DRKS00005363). Both adults and children were eligible for this analysis and were evaluated. Consistent with the Helsinki Declaration and its subsequent amendments, consent to participate in the respective registries was obtained from all patients and legal guardians. Table 1 provides an overview of the treatment plan characteristics of the evaluated cases. Only case ID 6 was an adult.

**TABLE 1:** Characteristics of the proton treatment of the evaluated cases.

Patient ID	Localization	Prescribed dose (GyRBE)	Setup uncertainty (mm)	Range uncertainty (%)	Min. energy (MeV)	Max. energy (MeV)	No. of fields/ room/ optim.	WET range shifter (cm)
0	Thorax	50.4	5	3.5	100	152	2/G/I	7.4
1	Brain	54	3	3.5	100	147	2/F/S	7.4
2	Brain	54	3	3.5	110	152	2/G/I	5.1
3	Abdomen	14.4	5	5.0	100	191	3/G/I	7.4
4	Brain	54	3	3.5	105	156	2/F/I	7.4
5	Abdomen	21.6	5	5.0	104	184	4/G/I	7.4
6	H and N	70	3	3.5	100	203	5/G/I	7.4
7	H and N	23.4	3	3.5	100	175	2/G/S	7.4
8	Pelvis	45	5	3.5	102	149	2/G/S	7.4
9	Brain	54	3	3.5	108	160	2/G/I	5.1
10	Brain	50.4	3	3.5	110	178	2/G/S	5.1
11	Brain	54	3	3.5	110	163	2/G/I	7.4

*Note*: “F” = fixed‐beam (horizontal) treatment room, “G” = gantry room, “WET” = water equivalent thickness, “optim.” = optimization, S = single‐field uniform dose optimization, I = intensity‐modulated PT planned with multi‐field optimization.

Abbreviation: PT, proton therapy.

The patients were scanned for treatment planning with a Brilliance Big Bore CT (Philips Healthcare, Best, The Netherlands). The tube voltage was set to 120 kV. The slice thickness was 1 mm. Two kernels (“UB” for head scans and“B” otherwise) of the iDose4 iterative reconstruction were used. All patients were treated with the spot‐scanning technique in a treatment room of a ProteusPlus proton machine (IBA PT, Louvain‐la‐Neuve, Belgium). All fields involved a range shifter. The treatment plans under evaluation were the clinical ones, which had been established in RayStation (RaySearch Laboratories, Stockholm, Sweden). Multi‐field treatment plans were robustly optimized according to the Minimax approach implemented in RayStation (version 12A).^14^ The robustness parameters fed into the optimizer concerned CTVs with a 3 mm isotropic set‐up uncertainty in the cranium and 5 mm elsewhere combined with a density uncertainty of 3.5%, which was increased to 5% when at least one field traversed the patient couch (Table 1). Three patients were planned with a single‐field uniform dose technique and multi‐field optimization otherwise. The final dose distributions were re‐calculated in a research implementation of RayStation (version 11B) on a 1 mm grid with the Monte‐Carlo engine requiring <0.5% statistical uncertainty for the 50% dose level of each field.

In order to determine the spatial resolution of the planning CT,^15^, ^16^ the quality assurance phantom of the vendor of the scanner was used, which contains a copper wire with 0.18 mm diameter embedded in a water cylinder of 20 diameter. The line‐spread function (LSF) was determined by averaging over the LSF of nine transversal directions. The spatial resolution will also be reported as FWHM of the LSF. In order to estimate the noise level of the clinical plans and to assess experimentally the influence of slice thickness and the volume CT dose index ${\text{CTDI}}_{\mathrm{vol}}$ on the noise level, spiral scans of an anthropomorphic phantom (ATOM, Computerized Imaging Reference Systems [CIRS], Inc.) representing a 5‐year‐old child (type 705D) were performed with the clinical virtual simulator CT described above. The clinical acquisition protocols according to scans of patients ID 02, ID 03, and ID 08 were used as they agreed within 2 years with the nominal age of the phantom. For the low‐dose scan of the brain case (ID 02) the ${\text{CTDI}}_{\mathrm{vol}}$ was reduced down to the machine limits by a factor of 7.3. Because the clinical protocols of the other localizations were already close to the machine limit, the ${\text{CTDI}}_{\mathrm{vol}}$ was increased by a factor of 11 to study the effect on noise. The noise in terms of standard deviation of the CT numbers was determined within homogenous regions of the brain, the thorax, the pelvis and the abdomen.

CT image sets were processed outside RayStation with Python scripts (version 3.9, scripts available for download at zenodo (DOI: 10.5281/zenodo.14039122)) and re‐imported into RayStation afterwards. Lower resolutions were mimicked by two types of three‐dimensional filters as introduced in Section 2. The first one was a rect‐filter with widths between 2 and 50 mm in steps of 2 mm (for widths <10 mm) and 4 mm (for widths ≥10 mm). The second filter was of Gaussian type ($\exp(-x^{2}/\left(2\sigma^{2}\right))$) with a cut‐off at 4σ at each side. The used filter widths ranged between 1.2 and 37.6 mm (FWHM). They comprised regular steps with spacings of 1.2 mm (for widths <4.7 mm) and 2.4 mm (for widths between 4.7 and 23.5 mm) and discrete steps of 28.2 and 37.6 mm. This smearing required an expansion of the external contour at interfaces to air. The external was adapted to the image set resulting from the broadest filter. Similar to ref. 17, a zero‐mean spatially uncorrelated Gaussian noise (standard deviation between 9 and 192 HU) was added to the reconstructed clinical CT scans. Rather high noise levels were considered to cover the imaging through secondary particles induced by the applied protons of a treatment fraction as shown, for example, in ref. 8. As the focus of this study is the influence of the resolution on the dose calculation, the evaluation with the addition of noise was restricted to three cases with tumors located in the thorax, brain, and pelvis. Because the noise was added after applying the smoothing filter, the noise level remained independent of the filter used.

After the dose calculation on the modified LR‐CT image sets, a global gamma‐index test was performed comparing the clinical dose distribution with the one on the deteriorated CT. The corresponding internal scripting function in RayStation^18^ was used with a 1 mm/1% criterion and a 10% dose threshold.^19^, ^20^ The gamma test was applied to all voxels of within the external contour and additionally restricted to voxels of the planning target volume (PTV).

The usage of LR‐CTs in a clinical workflow also requires that the dosimetric characteristics of perturbed positioning and anatomical scenarios are not influenced by the degradation of the IQ. This aspect is not covered by the theory of Section 2. Particularly, the inclusion of set‐up variations in the low‐pass filtering of the CTs could potentially alter the robustness budget in terms of set‐up shifts of the treatment plans on the LR‐CTs. Consequently, the in‐silico study also evaluated the dependence of robustness tests on the IQ of the underlying CT. In line with the clinical procedures in our institution, the Dutch robust evaluation scheme was adopted.^21^ Using the “robust evaluation” module in RayStation, six shifts of the patient position along the cardinal axes combined with two mass density perturbations were created. Evaluating the worst‐case scenario and the voxel‐wise minimum scenario, $V_{95\%}$[CTV] (with a minimum of 98%) served as a test for target coverage. A test with higher sensitivity was also included. It determined the dose D for which 50% of the perturbed scenarios fulfill $V_{D}\left[\mathrm{CTV}\right)]>98\%$. In this way, the influence of degraded IQ is reported as a bias of D. The tolerance doses of OAR, which could be potentially exceeded, were also regarded. Owing to the high computational demands, the evaluation of perturbed scenarios was limited to six patients.

## 4 RESULTS

The FWHM of the LSF, which describes the resolution of the clinical x‐ray CT scanner, was 1.38(±0.07) mm for the B‐type reconstruction filter and 1.53(±0.03) mm for the UB‐type filter (Table 2). The noise levels of the clinical protocols applied to a scan of an anthropomorphic phantom are shown in Table 3. The deviation of a square‐root relationship of noise of CT numbers when changing between 1 mm slices and 2 mm slices (Equation 15 in Section 5), which corresponds approximately to a boxcar‐type low pass filtering, was 4% (12%) on average (maximum). When upscaling or downscaling the mAs setting of the CT protocols, the deviation of a square‐root relationship between ${\text{CTDI}}_{\mathrm{vol}}$ and noise (Equation 15) was 16% (31%) on average (maximum).

**TABLE 2:** Results of the measurements of the spatial resolution (MTF50 und MTF10) of the Philips Brilliance Big Bore CT using B‐type and UB‐type reconstructions and comparison with previous studies (Arisyi et al.^22^ and Tomic et al.^23^).

	This study		Arisyi et al.		Tomic et al.	
Scanner (model)	Brilliance Big Bore		Brilliance		Brilliance Big Bore	
Filter	UB	B	UB	B	UB	B
kV	120	120	120	120	120	120
mAs	350	168	300	300	274	103
FOV (mm)	200	200	200	200	240	240
Pitch	0.56	0.81	0.56	0.56	0.44	0.82
Slice thickness (mm)	1	1	2	2	5	5
MTF50 (${\text{cm}}^{-1}$)	4.1	4.2	3.4	4.2	3.2	2.8
MTF10 (${\text{cm}}^{-1}$)	7.4	8.0	6.3	7.8	5.7	4.8

Abbreviations: CT, computed tomography; FOV, field of view.

**TABLE 3:** Noise level of CT numbers in terms of standard deviation (“std. dev.”) for pediatric protocols, which are clinically used for virtual simulation.

		Pixel			CT‐number
	Current	size		${\text{CTDI}}_{\mathrm{vol}}$	std. dev.
Localization	modulation	(mm)	(mAs)	(mGy)	(HU)
Brain	No	0.68	220	26.2	6
Abdomen	Yes	1.17	30	2	11.5
Thorax	Yes	1.17	30	2	8.1
Pelvis	Yes	1.17	30	2	12.8

Abbreviations: CT, computed tomography; HU, Hounsfield unit.

**FIGURE 1:**
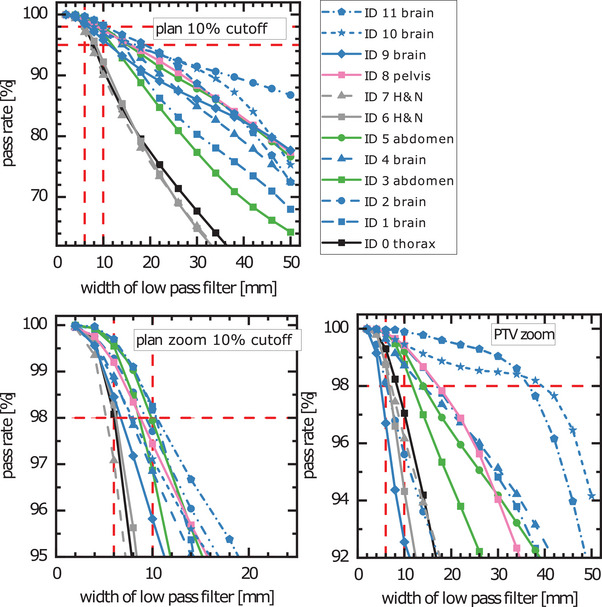
Pass rate of the gamma test (1 mm/1%, 10% dose threshold) as a function of the width of the low pass filter. A rect‐type filter kernel was used to mimic an x‐ray CT with low spatial resolution. The horizontal red dashed line indicates the required pass rate of 98%. Vertical dashed lines indicate twice the value of the respective set‐up margin, which was used in robust optimization and indicates the minimum value for an acceptable distortion of the dose distribution according to theoretical considerations. Top (bottom) left: statistics for all voxels within the external (zoomed). Bottom right: only voxels within the PTV have been considered. CT, computed tomography; PTV, planning target volume.

**FIGURE 2:**
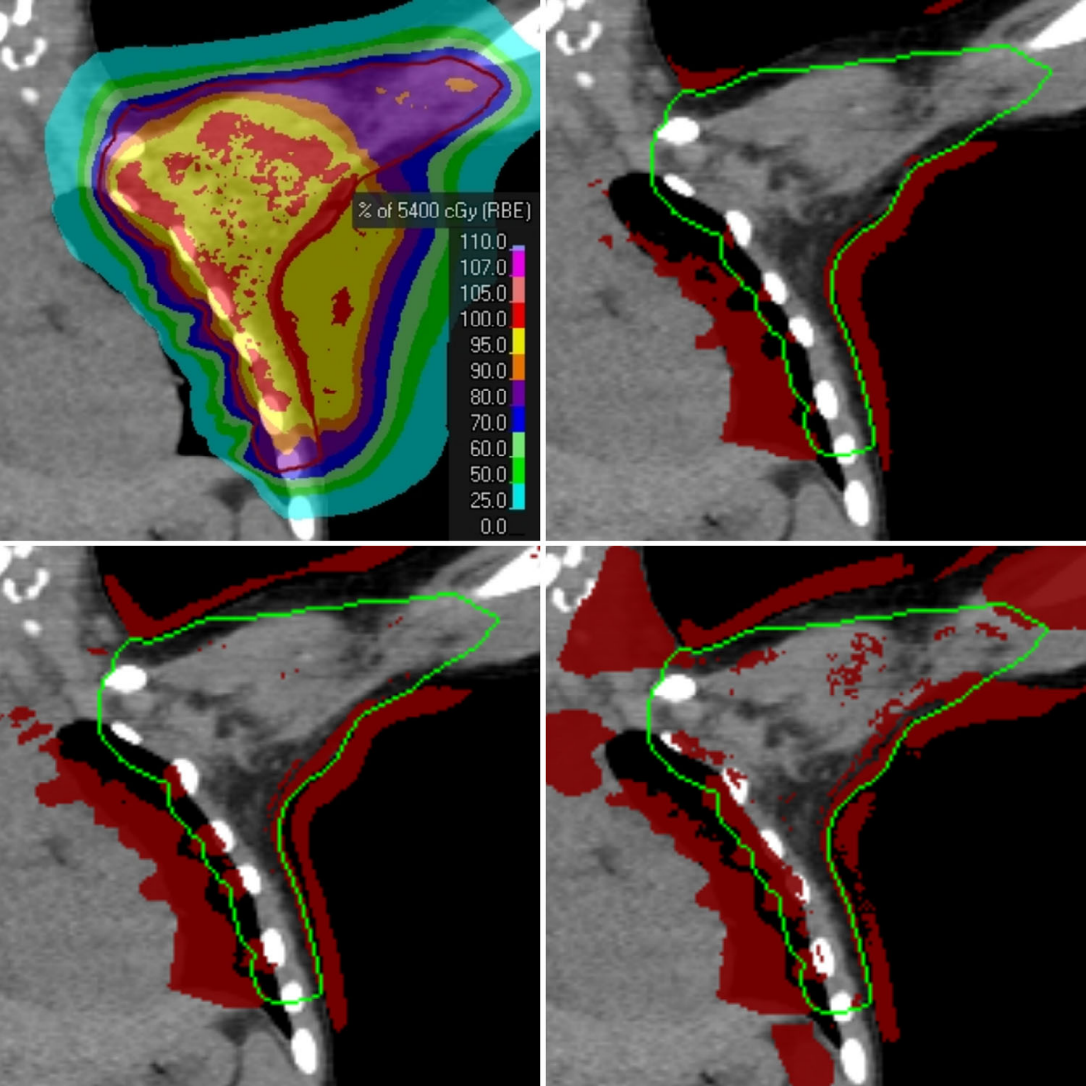
Example of patient ID 0, which had a PTV located at the chest wall: dose distribution (top left, PTV = red contour) and results of the gamma index test (bottom left, right; PTV = green contour) visualized in coronary planes. The underlying CT image is the clinical one for all sub‐figures. The red overlay indicates voxels with failed gamma index test. These test results were obtained on low‐pass filtered image sets with a Gaussian of width (FWHM) 4.7 mm (top right), 8.2 mm (bottom left), 18.9 mm (bottom right). CT, computed tomography; FWHM, full‐width‐at‐half‐maximum; PTV, planning target volume.

The pass rate of the gamma test as a function of the width of a rect‐type filter with the patient ID as a parameter is depicted in Figure 1 (top, bottom left). A gamma pass rate of 98% is achieved for kernel widths ranging between 4 and 10 mm. Restricting the gamma test to voxels of the PTV (Figure 1 [bottom right]), results generally in higher pass rates. The maximum acceptable kernel width is 4 mm, too. The plan of the thoracic tumor (patient ID 0) is the least robust one against deterioration of the spatial resolution. This is visualized in Figure 2, which indicates that failed voxels are mainly located at the interface between soft tissue and low‐density volumes of the virtual patient. Furthermore, there is a head‐and‐neck plan (ID 7) with a clear drop of the pass rate beyond 4 mm kernel width when all voxels are considered Figure 1 (top, bottom left). For two brain cases (ID 10 and 11), kernel widths up to ≈34 mm are acceptable regarding the dose computation in the PTV. Table 4 summarizes the acceptable kernel widths.

**TABLE 4:** Maximum width of boxcar filter (in mm) which fulfills the requirements on the gamma pass rate (>98% with 1mm/1%, 10% dose threshold criterion).

Localization		Max. kernel		
		width		
Localization	ID	for body	for PTV	Robustness effect of CT low‐pass filtering
Brain	1	10	14	Negligible for CTV, no adjacent OAR
	2	8	6	Not tested
	4	**6**	10	Negligible for CTV and periventricular region
	9	**6**	**4**	Not tested
	10	**6**	38	Not tested
	11	10	34	Not tested
H and N	6	6	**6**	Negligible for CTV, myelon (≤1.5%) and brain stem
	7	**4**	**6**	Negligible for CTV, myelon and eye lens (≤2.5%)
Abdomen	3	**8**	**10**	Not tested
	5	**8**	**10**	Negligible for CTV, liver and left kidney
Thorax	0	**4**	8	$D_{\mathrm{avg}}\left[\mathrm{left}\;\mathrm{lung},\;\mathrm{worst}\;\mathrm{case}\right]$ drops ≈3% per 10 mm filter width
Pelvis	8	**6**	6	Negligible for CTV and intestine

*Note*: Bold kernel widths indicate the minimum value per localization. For OAR, percentage dose deviations of the perturbed scenario are based on a 4 mm boxcar filtering with maximum deviation to the respective evaluation on the clinical treatment plan. Values are only reported if deviations >1%. $D_{\mathrm{avg}}$: average organ dose.

Abbreviations: CT, computed tomography; CTV, clinical target volume; OAR, organs‐at‐risk; PTV, planning target volume.

The gamma pass rates between dose distributions on LR image sets with boxcar filter were compared to those smoothed with Gaussian filter. A factor of $1/\sqrt{12}$ was used to scale the width of the boxcar filter to the standard deviation of the normal distribution, which could be regarded as untruncated due to the rather large cut‐off values applied. The width of boxcar‐type filters was smaller by 6% on average (7% standard deviation) than the widths of the corresponding Gaussian filters, which led to the same respective gamma pass rate (≥95%). The filter kernel types were regarded as equivalent due to the small difference. To keep the presentation of the results concise, the following presentation of each result is limited to one kernel type.

**FIGURE 3:**
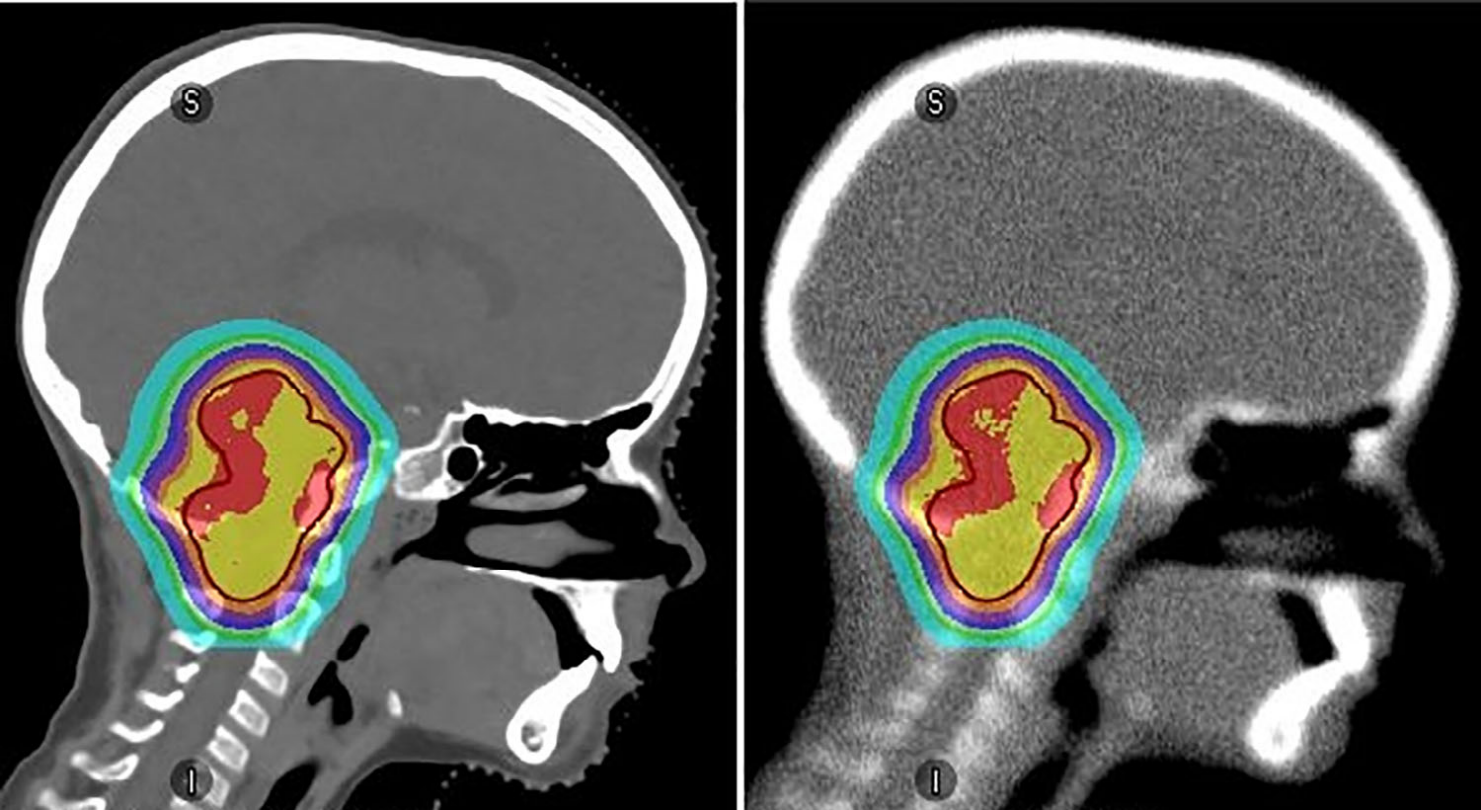
Exemplary sagittal dose distributions (colorwash overlay with the same color code as Figure 2) of patient ID 2. The red contour indicates the PTV. Left: planning CT overlaid with clinical dose distribution. Right: Modified CT (folded with Gaussian of width = 4.7 mm [FWHM] and applied noise of σ = 48 HU). The dose distribution computed on this CT is equivalent to the left one with a gamma pass rate of 98.9% (1 mm/1%). CT, computed tomography; FWHM, full‐width‐at‐half‐maximum; HU, Hounsfield unit; PTV, planning target volume.

Figure 3 gives a visual impression of the quality of the CT, which is necessary to accurately compute the dose distribution. The dose distribution computed on the low‐pass filtered and noise‐added CT (right) is clinically equivalent to the one on the clinical CT, that is, the gamma pass rate is larger than 98%.

According to the robustness evaluation, $V_{95\%}$[CTV] of the worst cases scenarios and voxel‐wise minimum dose distributions changed typically on a per mille level if boxcar filters up to 26 mm were applied. The value of D, for which 50% of the perturbed scenarios fulfill $V_{D}\left[\mathrm{CTV}\right]>\;98\%$, changed typically by a few per mille. Table 4 (right column) includes an overview of the dosimetric deviations identified in the robustness tests. The dosimetric changes of the perturbed plans were negligible within the kernel width limits reported above for the CTV coverage. In total, 10 OARs, which were either situated in the high‐dose region or which had dose‐volume parameters close to the tolerance dose, were evaluated. The changes of the dose parameters of the most extreme perturbed scenarios after filtering with ≤4 mm wide boxcar filters were ≈2% for the myelon (ID 6), eye lens (ID 7), and lung (ID 0) and below 1% for all other OARs.

**FIGURE 4:**
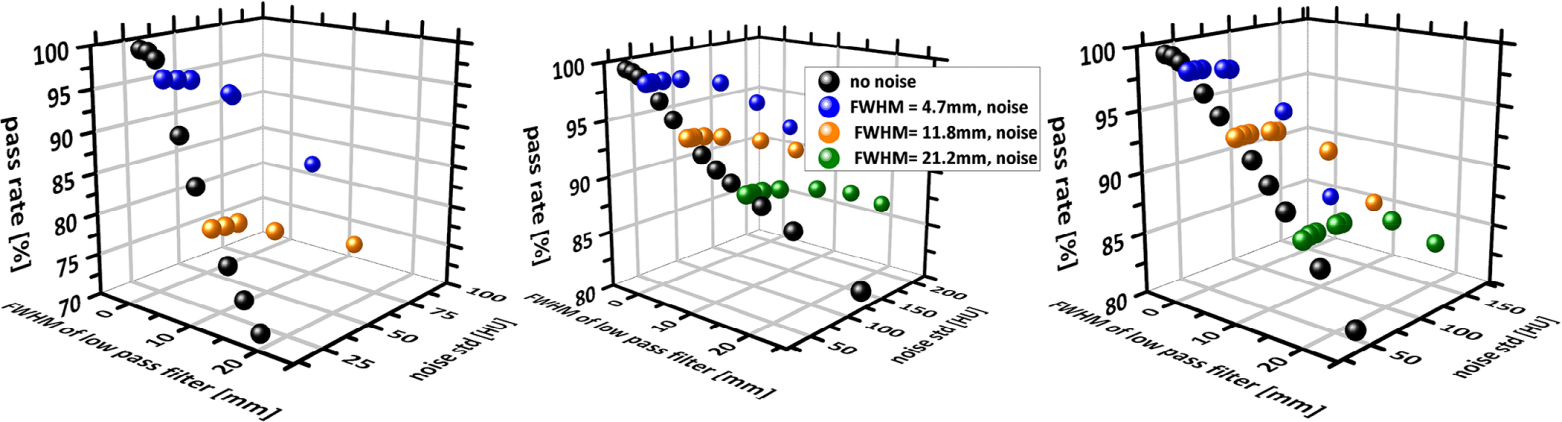
Pass rate of the gamma test as a function on the filter width (Gaussian type, denoted by the FWHM) and the level of the added noise. Left: patient ID 0 (thorax). Noise level of planning CT: 2.3 HU (std. dev.). Middle: patient ID 2 (brain), 6.0 HU (std. dev.). Right: patient ID 8 (pelvis), 9.6 HU (std. dev.). CT, computed tomography; FWHM, full‐width‐at‐half‐maximum; HU, Hounsfield units.

Figure 4 shows the gamma pass rate as a function on the filter width and the level of the added noise. For noise increases of up to about 10–20 HU, the pass rate values of the low‐pass filtered CTs remain constant or show a weak dependence on the level of additional noise. By and large, this finding is independent of the width of the low‐pass filter. While for patient ID 0 the gamma pass rate drops below 98% for a 4.7 mm (FWHM) Gaussian filtering in combination with the addition of noise with a standard deviation of 16 HU, an acceptable dose distribution can be computed with additional noise up to about 96 HU (43 HU) and the same low‐pass filtering for case ID 02 (ID 08). This corresponds to a noise increase of a factor of 16 (4.5) for ID 02 (ID 08).

## 5 DISCUSSION

The measured FWHM values of the LSF of 1.4–1.5 mm of the clinical planning CT were rated as comparable with previous findings as shown in Table 2. The measured FWHM also agrees with the corresponding value of 1.45±0.1 mm, which is quoted in the manual of the phantom for a quality assurance head scan with a field‐of‐view of 100 mm. The differences of MTF10 and MTF50 to the respective quantity from two previous studies (Table 2) were smaller than the respective deviations between these studies. The filter width characterizing the drop‐off of the pass rate is about a factor of two larger than the width of the LSF. Therefore, the exact values of the measured MTF50 and MTF10 (Table 2) are of minor relevance.

By and large, the limit for the image resolution motivated in Section 2 corresponded to the drop‐off of the gamma pass rate observed of the in‐silico study (Figure 1). The position of the decline of the gamma pass rate (Figure 1) could also be influenced by robust optimization concerning the density uncertainty, which has approximately the same spatial extent as the set‐up uncertainty. Similarly, the distortion of the dose distribution might depend on the width of the proton pencil beam, which is on a scale of ≈10 mm in air and could approximately take on twice the value when traversing the patient.

The theory of Section 2 applies to external RT in general, while the in‐silico study was restricted to PT. Each dose voxel $D_{i}$ depends on the voxels of the patient model $c_{1},c_{2},\text{…},c_{N}$ in a nonlinear manner, which is linearized in the mathematical derivation by a Taylor series. The respective contributions from $c_{k}$ of all pixels k are added. The derivative and higher‐order terms comprise variations of $c_{k}$ and $D_{i}$ in the percentage range. It is expected that these random contributions of all voxels compensate each other. Thus, the validity of Equation (12) is expected to correlate with the number of voxels $c_{k}$ which influence $D_{i}$. Above mentioned nonlinearity is not restricted to the dose engine but applies already to the CT calibration curve, which transforms from the CT number to electron or mass density, and to the logarithmic operation on projections in the x‐ray CT discussed below. The mathematic idea to represent alternative anatomical representations by linear combinations of the underlying image sets is not novel. This approach has been employed for 4D‐CT data set by ref. 24. The theory of the current study provides retrospectively a mathematic justification.

The accuracy of the dose calculation on LR‐CTs was better for most of the targets in the brain and in the abdomen than for targets of the thorax and head and neck (Figure 1). Table 4 indicates the supposed relevance of heterogenous localizations. This could be explained by the need for explicit robust planning, which was assumed in the theoretical considerations (Section 2), only in heterogenous anatomical regions. Similarly, the trend of higher gamma pass rates for voxels within the PTV (Figure 1 bottom right) compared to the irradiated volume (Figure 1 top, bottom left) might be explained by the restriction of the robust optimization to the target volume. We consider the maximum dose deviations of ≈2% in the worst case and voxelwise minimum perturbed scenarios (Table 4) induced by low‐pass filtering to be clinically acceptable. This finding and the gamma pass rates (Figure 1 and Table 4) indicate that a generic boxcar kernel width of 4 mm (Table 4), which corresponds approximately to a 2.7 mm (FWHM) wide Gaussian‐shaped PSF, appears to be generally acceptable. Because the boxcar filtering led to a stronger decrease of the gamma pass rate than Gaussian filters with the same standard deviation, the results of Figure 1 and Table 4 can be interpreted as a conservative estimation of the acceptable spatial resolution. Because the dose criterion of the gamma‐index test (1%) is only a factor of 2 larger than the statistical uncertainty of the Monte‐Carlo simulation (0.5%), the presented sensitivity to image degradation could be overestimated in the current study, too.

The insights of this study can be used to roughly estimate the potential for sparing of the x‐ray exposure E, which is given for example, by ${\text{CTDI}}_{\mathrm{vol}}$. The noise, which is described by the variance “Var”, can be described for tomographic images with penetrating photons by the fundamental relation^25^:

(15) $$\mathrm{Var}\;c\left(x\right)\propto \frac{1}{E\Delta \xi^{4}}$$

Here, ξ is the width of the PSF. For instance, it is the FWHM of the quadratic addition of the width of p and the initial resolution of $\tilde{c}$ in Equation (1). For ease of discussion, the easiest expression was used in Equation (15), which, for example, does not distinguish between resolution in lateral and longitudinal direction.^26^ The width of the PSF of the used x‐ray scanner of 1.4–1.5 mm would be broadened by a factor of 2.1 when folded with a boxcar‐type kernel width of 4 mm. In this way, the x‐ray exposure E could be reduced by a factor of 20 according to Equation (15). Considering the results of phantom scans and reconstructions with varying slice thickness (Section 4), this estimation has an uncertainty of about 10%. Figure 4 indicates that for all simulated spatial resolutions the addition of noise does not decrease the gamma pass rate to a certain extent, that is, the addition of a limited amount of noise is independent of the lowering of the spatial resolution. If the low‐pass filtering is combined with the addition of noise (Figure 4), then one or two orders magnitude of additional dose sparing would be theoretically feasible for some patients according to Equation (15).

However, this theoretical dose sparing potential might refer to unrealistic scanning concepts. For example, the x‐ray CT scanner of our institute allows for a dose reduction by a factor of seven in brain protocols, which is in line with the reductions of the x‐ray exposure down to 10% reported in ref. 10. A better dose sparing might be achieved with other CT scanner models, for example, by incorporating a tin filter. Using this technique for ultra low‐dose CT scans for attenuation correction in PET, Mostafapour et al. found acceptable quality of the activity images when reducing the x‐ray exposure to 2% of the nominal value.^27^ As this protocol was, however, associated with stronger streak artifacts related to long x‐ray paths through the body, a 10% reduction of the exposure appeared to be a pragmatic approach.^27^ In general, ultra‐low x‐ray current acquisition protocols in conjunction with LR reconstruction might be technically feasible. We conjecture that this mode has not been considered yet by the vendors, because CT scanners are not designed for the applications investigated in the current study. The results might motivate the inclusion of such a mode into virtual simulators. The discussed studies^10^, ^27^ refer to CT scanners with integrating‐mode detectors. Owing to their low electronic noise, photon‐counting mode CTs, which became recently commercially available, are expected to mitigate photon starvation artefacts thereby pushing the limits for ultra‐low dose imaging.^28^ Furthermore, the results directly apply to γCTs from the joint reconstruction of ToF‐PET scans,^8^, ^29^ which is low‐statistics, LR tomography compared to x‐ray CT. Generally, the x‐ray exposure expended for virtual simulation and patient positioning is a non‐negligible contribution to the overall out‐of‐field dose in PT.^30^ Thus, it makes sense to devise measures to reduce the x‐ray exposure, especially for pediatric patients. Strong reductions of the x‐ray exposure have also been investigated for cone‐beam CT‐based image guidance in RT of pediatric patients.^31^ Besides the addition of noise in the projection domain, the dose reduction was simulated by decimating the angular projections.^32^ The latter processing is similar to the one of the current work, which, in contrast, also reduced the bandwidth.

The applied low‐pass filtering in the image domain (Section 3) might not reflect the data processing within photon‐based CT scanners, where a rebinning of acquired projections in sinogram space could be performed before logarithmic operation. For CT scanners with low‐noise, high‐resolution detectors, a resampling to larger voxels is recommended at a later stage of reconstruction^33^, ^34^ in agreement with the methodology of the current study. This study included filter kernels with a truncated PSF of Gaussian shape. This shape is approximately preserved when folding with the PSF of the planning CT (Table 2). This is not an exact but a reasonable PSF model of x‐ray CT scanners.^35^ Because the results exhibit a negligible dependence on the filter type, little impact is expected if other types of filter kernels, for example, triangular‐shaped ones, would be used. A further limitation of the generic approach of the study is that a white noise distribution was assumed thereby simplifying the distribution of noise in x‐ray CT scanning.^10^ While the simulation of the degradation of IQ with linear filters is a rough approximation for photon‐based CTs, its generic nature makes it applicable for all type of tomographic modes in leading‐order approximation. For example, LR image sets are also expected for synthetic CTs generated in the frame of MRI‐based, online adaptive RT with fast MRI sequences, which entail a coarser spatial sampling. The results are also relevant for the design of proton CTs.

A limitation of the evaluation of the dose computation on image sets, which were low‐pass filtered and degraded with additional noise, is the restriction to three cases. A further restriction is the assessment of the dose computation and not optimization as in ref. 10. A further limitation is the size of the evaluated patient cohort (N=12). If a general limit per localization was based only on an in‐silico study, then many more cases would have to be analyzed. For replanning in an adaptive treatment scheme such a general limit is not necessary, because the required spatial resolution for verification scans could be evaluated individually using the planning CT.

In the PT planning workflow, x‐ray CTs are used for contouring and dose computation. The former application has different requirements than the latter one, for example, low noise and good soft tissue contrast. Considering that modern PT planning is based on multimodal imaging, this can, for example, be achieved with MRI thereby relaxing the IQ requirements on x‐ray CTs. The low dose CTs also need to be registered to other image sets. Recent studies of cone beam CT guided RT demonstrate the robustness of registration against decimation of the number of cone beam projections.^31^, ^36^

## 6 CONCLUSIONS

Dose distributions of PT plans calculated retrospectively on smoothed CTs remained essentially undisturbed for boxcar‐type smoothing filter widths below 4 mm, which approximately corresponds to the limit derived from theoretical considerations. Equivalent results were obtained with a Gaussian kernel with the same standard deviation. There was a differentiated behavior with regard to the anatomical position of the tumor. In particular, targets in the brain, abdomen, and pelvis were robust to low‐resolution CTs. The relaxed requirements on spatial resolution of the underlying image set shows a theoretical dose saving potential of a factor of 20 for tomographic scanning. It was demonstrated that noise could be added to a certain degree to smoothed CTs with limited distortions of the calculated dose distributions. In perspective, these findings could be incorporated into an adaptive PT workflow in order to reduce the dose burden of verification imaging.

## CONFLICT OF INTEREST STATEMENT

The authors have no relevant conflicts to disclose.

## Data Availability

Experimental data files and evaluation scripts are available at: 10.5281/zenodo.14039122.
